# The role of histological subtype in hormone receptor positive metastatic breast cancer: similar survival but different therapeutic approaches

**DOI:** 10.18632/oncotarget.8838

**Published:** 2016-04-19

**Authors:** Dorien Lobbezoo, Wilfred Truin, Adri Voogd, Rudi Roumen, Gerard Vreugdenhil, Marcus Wouter Dercksen, Franchette van den Berkmortel, Tineke Smilde, Agnes van de Wouw, Roel van Kampen, Johanna van Riel, Natascha Peters, Petronella Peer, Vivianne C.G. Tjan-Heijnen

**Affiliations:** ^1^ GROW, School for Oncology and Developmental Biology, Maastricht University Medical Center, Maastricht, The Netherlands; ^2^ Department of Internal Medicine, Máxima Medical Center, Veldhoven, The Netherlands; ^3^ Department of Surgery, Máxima Medical Center, Veldhoven, The Netherlands; ^4^ Department of Internal Medicine, Atrium-Orbis, Heerlen, The Netherlands; ^5^ Department of Internal Medicine, Jeroen Bosch Hospital, Den Bosch, The Netherlands; ^6^ Department of Internal Medicine, VieCuri Medical Center, Venlo, The Netherlands; ^7^ Department of Internal Medicine, Atrium-Orbis, Sittard, The Netherlands; ^8^ Department of Internal Medicine, St Elisabeth Hospital, Tilburg, The Netherlands; ^9^ Department of Internal Medicine, St Jans Hospital, Weert, The Netherlands; ^10^ Department for Health Evidence, Radboud University Medical Center, Nijmegen, The Netherlands

**Keywords:** metastatic breast cancer, histology, invasive lobular carcinoma, invasive ductal carcinoma, treatment

## Abstract

**Introduction:**

This study describes the differences between the two largest histological breast cancer subtypes (invasive ductal carcinoma (IDC) and invasive (mixed) lobular carcinoma (ILC) with respect to patient and tumor characteristics, treatment-choices and outcome in metastatic breast cancer.

**Results:**

Patients with ILC were older at diagnosis of primary breast cancer and had more often initial bone metastasis (46.5% versus 34.8%, *P* = 0.01) and less often multiple metastatic sites compared to IDC (23.7% versus 30.9%, *P* = 0.11). Six months after diagnosis of metastatic breast cancer, 28.1% of patients with ILC and 39.8% of patients with IDC had received chemotherapy with a longer median time to first chemotherapy for those with ILC (*P* = 0.001). After six months 84.8% of patients with ILC had received endocrine therapy versus 72.5% of patients with IDC (*P* = 0.0001). Median overall survival was 29 months for ILC and 25 months for IDC (*P* = 0.53).

**Materials and Methods:**

We included 437 patients with hormone receptor-positive IDC and 131 patients with hormone receptor-positive ILC, all diagnosed with metastatic breast cancer between 2007–2009, irrespective of date of the primary diagnosis. Patient and tumor characteristics and data on treatment and outcome were collected. Survival curves were obtained using the Kaplan-Meier method.

**Conclusions:**

Treatment strategies of hormone receptor-positive metastatic breast cancer were remarkably different for patients with ILC and IDC. Further research is required to understand tumor behavior and treatment-choices in real-life.

## INTRODUCTION

The two most frequent histological subtypes of breast cancer are invasive ductal carcinoma (IDC) and invasive lobular carcinoma (ILC), with IDC comprising 75–80% and ILC 5–15% of all breast cancer cases. ILC is being associated with larger tumor size at presentation, more bilateral and multifocal involvement and with a different pattern of metastatic spread compared with IDC [1, 2]. Furthermore, ILC is more often HR-positive, HER2-negative, with lower S-phase fraction and less often positive for the tumor suppressor gene p53, compared with IDC [1]. Also, treatment response is known to be different. In a combined analysis on a number of retrospective series, the pathological complete response rate of neo-adjuvant chemotherapy was significantly lower in ILC (1.7%) than in IDC (11.6%) [3]. In the adjuvant setting, retrospective data suggest a higher efficacy of endocrine therapy for ILC than IDC [4]. More recently, a large retrospective study strongly suggested that HR-positive breast cancer patients with ILC do not seem to benefit from adjuvant chemotherapy in addition to endocrine therapy [5].

Whether these differences have a prognostic impact is controversial. Some studies found no effect of histology on survival [1, 6, 7], others found a better prognosis for patients with ILC compared to those with IDC [8–10] and some found a change in prognosis over time with a better prognosis for ILC during the first years of follow-up and a worse prognosis during later years [2, 11].

So far, studies on histological subtypes consider early breast cancer and not metastatic breast cancer. The aim of this study was to describe the differences between IDC and ILC with respect to patient and tumor characteristics, treatment-choices and outcome in metastatic breast cancer. In order to account for the effect of the hormone receptor (HR) on outcome and treatment-decision making, we only included patients with HR-positive breast cancer.

## RESULTS

### Patient characteristics

Of the 568 patients with HR-positive metastatic breast cancer, 23% had (mixed type) ILC (131 patients), hereafter referred to as ILC and 77% had IDC (437 patients) (Table 1).

**Table 1:** Baseline characteristics of HR+ metastatic breast cancer divided by histological subtype

Characteristics	Invasive ductal carcinoma *N*= 437		Invasive lobular and mixed carcinoma *N* = 131		*P*
	No	%	No	%	
**Age at primary diagnosis**					
Median (range)	58 (25–91)		62 (36–89)		0.03
< 50 years	124	28.4	28	21.4	0.11
≥ 50 years	313	71.6	103	78.6	
**Histological grade of primary tumor**					
SBR 1	41	13.4	17	19.8	0.11
SBR 2	157	51.5	48	55.8	
SBR 3	107	35.1	21	24.4	
Unknown	132		45		
**Primary tumor stage**					
T1	157	48.2	29	30.2	< 0.001
T2	150	46.0	51	53.1	
T3	12	3.7	9	9.4	
T4	7	2.1	7	7.3	
Unknown	111		35		
**Regional lymph node stage**					
N0	141	43.1	34	34.0	0.005
N1	110	33.6	40	40.0	
N2	50	15.3	8	8.0	
N3	26	8.0	18	18.0	
Unknown	110		31		
**HER2 status of primary tumor**					
Positive	67	15.3	10	7.6	0.02
Negative	370	84.7	121	92.4	
**Prior adjuvant endocrine therapy**					
Yes	228	52.2	76	58.0	0.24
No	209	47.8	55	42.0	
**Prior adjuvant chemotherapy**					
Yes	135	30.9	40	30.5	0.94
No	302	69.1	91	69.5	
**Prior adjuvant targeted therapy**					
Yes	18	4.1	1	0.8	0.06
No	419	95.9	130	99.2	
**Metastatic-free interval (MFI)**					
Median (range), months	38.7 (0–234)		32.3 (0–321)		0.58
De novo	81	18.5	26	19.9	0.1
MFI ≤ 24 months	64	14.7	28	21.3	
MFI > 24 months	292	66.8	77	58.8	
**Initial site of metastasis**					
Bone	152	34.8	61	46.5	0.01
Visceral	114	26.1	30	22.9	0.50
Brain	8	1.8	3	2.3	0.74
Skin and lymph nodes	28	6.4	6	4.6	0.44
Multiple*	135	30.9	31	23.7	0.11

Abbreviations; HR+ hormone receptor positive, *N* number, SBR Scarff Bloom Richardson, MFI metastatic-free interval * ≥ 1 of the aforementioned initial sites of metastasis.

Metastatic breast cancer patients with ILC were older at the time of primary breast cancer diagnosis, compared with patients with IDC (median age at diagnosis 62 years for ILC versus 58 years for IDC, *P* = 0.03). At initial presentation, patients with HR-positive ILC had larger tumors (T2-3, 62.5% versus 49.7%, *P* = 0.002) and slightly more node-positive disease (66% versus 56.9%, *P* = 0.10), but with less often a HER2 positive status (7.6% versus 15.3%, *P* = 0.02) and lower grade (not significant) compared with patients with HR-positive IDC. No differences were seen in use of adjuvant chemotherapy or endocrine therapy between the histological subtypes.

Bone was the most common initial site of distant metastasis in both HR-positive histological subtypes, although bone metastasis as initial site was more frequently observed for patients with ILC compared to those with IDC (46.5% versus 34.8%, *P* = 0.01). Fewer patients with ILC were diagnosed with multiple sites of distant metastasis compared to those with IDC (23.7% versus 30.9%, *P* = 0.11).

### Palliative systemic treatment

Median follow-up after diagnosis of metastatic disease was 37.1 months (range 5.2–54.6) with 239 patients (42%) alive at the end of the follow-up period.

**Figure 1:**
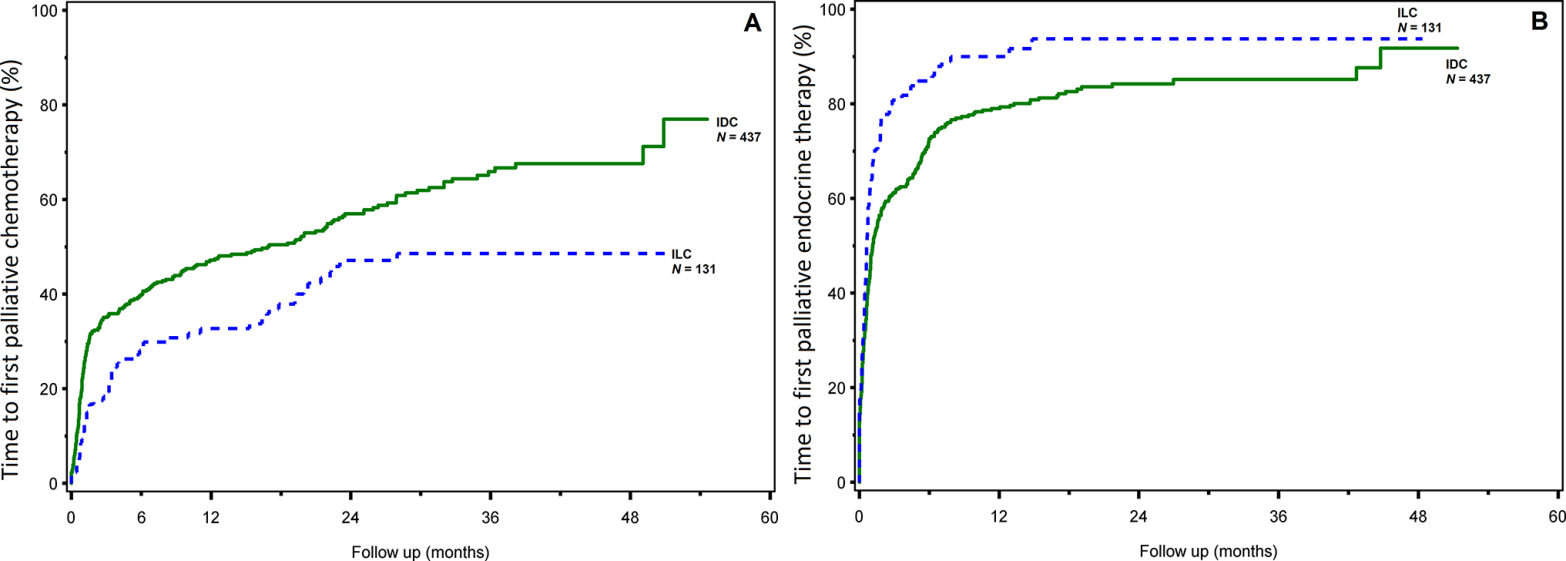
Time to first palliative chemotherapy (**A**) and endocrine therapy (**B**) by histological subtype.

Time between diagnosis of HR-positive metastatic breast cancer and start of palliative chemotherapy was significantly longer for patients with ILC (median not yet reached) compared with IDC (median 16.9 months, 95% Confidence Interval (CI) 9.6-22.3, *P* = 0.001) (Figure 1A). Six months after diagnosis of HR-positive metastatic breast cancer less patients with ILC had received palliative chemotherapy compared with IDC (28.1% versus 39.8% respectively).

Time between diagnosis of HR-positive metastatic breast cancer and start of palliative endocrine therapy was significantly shorter for patients with ILC (median, 0.6 months, 95% CI 0.5–0.8) compared with patients with IDC (median, 1.1 months, 95% CI 1.0–1.5, *P* = 0.0001) (Figure 1B). Six months after diagnosis of metastatic breast cancer 84.8% of patients with ILC had received palliative endocrine therapy compared with 72.5% of patients with IDC.

### Outcome

Median overall survival was 29.4 months (95% CI 22.5–36.6) for patients with HR-positive ILC and 25.4 months (95% CI 21.8–31.7) for patients with HR-positive IDC (*P* = 0.53).

In multivariable analysis for patients with ILC with palliative endocrine therapy and palliative chemotherapy as time-dependent covariates, early initiation of palliative chemotherapy was associated with an unfavorable survival (hazard ratio 2.8, 95% CI 1.7–4.6, *P* < .0001) compared to no palliative chemotherapy during the observation period. Conversely, early initiation of palliative endocrine therapy was associated with a favorable survival (hazard ratio 0.4, 95% CI 0.2–0.8, *P* = 0.005) compared to no palliative endocrine therapy during the observation period.

In multivariable analysis for patients with IDC, early initiation of palliative chemotherapy was associated with an unfavorable survival (hazard ratio 2.1, 95% CI 1.6–2.7. *P* < .0001) when compared with no chemotherapy, whereas early treatment with palliative endocrine therapy was not associated with survival (hazard ratio 0.9, 95% CI 0.6–1.2, *P* = 0.4).

### Residual survival: six months after diagnosis

**Figure 2:**
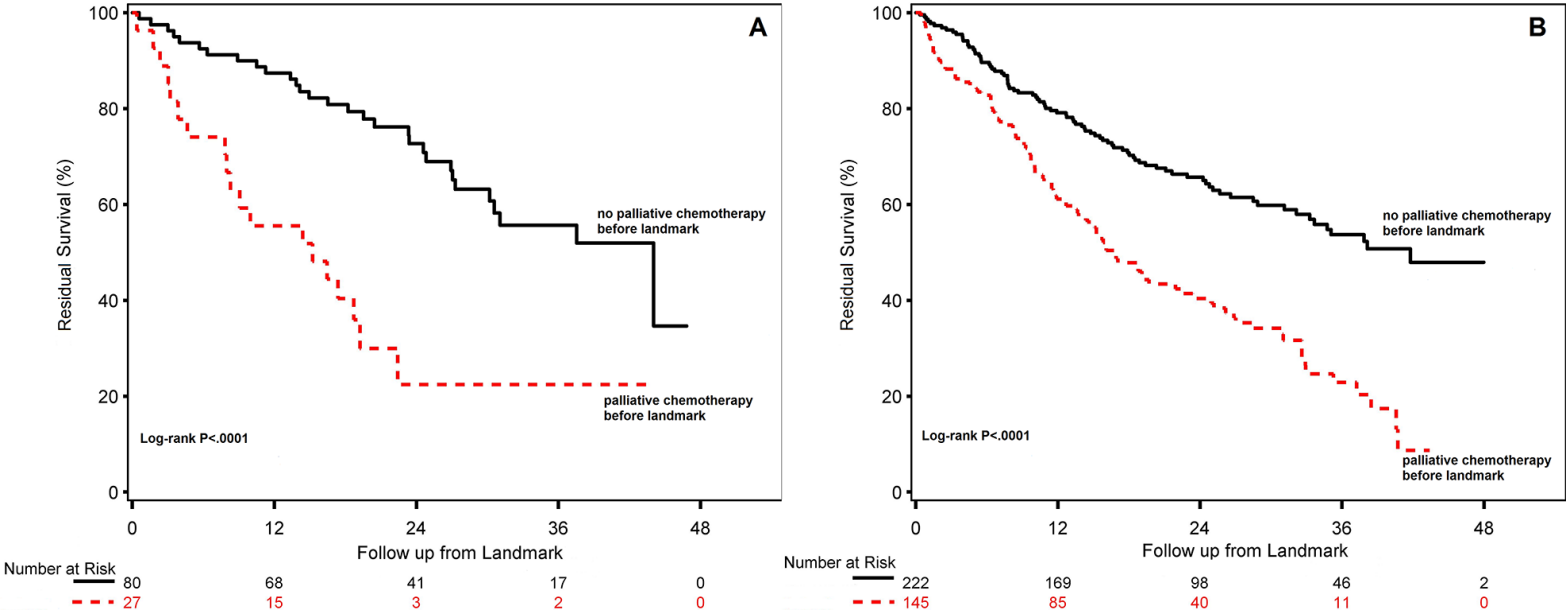
Residual survival for patients with HR-positive ILC (**A**) and HR-positive IDC (**B**) treated with or without any palliative chemotherapy during the first six months after diagnosis of metastatic breast cancer.

For ILC, the residual survival was significantly longer for patients not treated with palliative chemotherapy within the first six months after diagnosis of metastatic breast cancer (median 44.0 months, 95% CI 30.2-not yet reached) versus patients treated with palliative chemotherapy (median 15.2 months, 95% CI 7.8–19.2) (Figure 2A). For IDC, residual survival when not treated with palliative chemotherapy was 41.8 (95% CI 33.3-not yet reached) compared with 16.8 months (95% CI 13.6–22.5) for patients treated with palliative chemotherapy within the first six months after diagnosis of metastatic breast cancer (Figure 2B).

**Figure 3:**
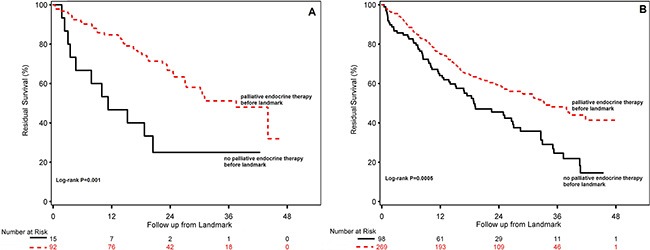
Residual survival for patients with HR-positive ILC (**A**) and HR-positive IDC (**B**) treated with or without any palliative endocrine therapy during the first six months after diagnosis of metastatic breast cancer.

Reversely, for ILC-patients with palliative endocrine treatment within the first six months after diagnosis of metastatic breast cancer residual survival was 37.5 months (95% CI 26.9-not yet reached) compared with 11.2 months (95% CI 3.0–20.4) for patients with HR-positive metastatic ILC without palliative endocrine therapy before the first six months (Figure 3A). For patients with HR-positive metastatic IDC treated with palliative endocrine therapy during the first six months, residual survival was 33.5 months (95% CI 25.7-not yet reached) compared with 18.9 months (95% CI 14.0-26.8) for patients with HR-positive metastatic IDC without palliative endocrine therapy in the first six months of metastatic disease (Figure 3B).

## DISCUSSION

To our knowledge, the role of histological subtype in palliative systemic treatment of metastatic breast cancer has not been studied before. In a cohort of 568 HR-positive metastatic breast cancer patients, we showed that patients with HR-positive ILC had more often bone and less often multiple sites as initial site of distant metastasis compared to those with HR-positive IDC. Six months after metastatic diagnosis, patients with HR-positive ILC had received significantly less often chemotherapy (28% versus 40%) and more often endocrine therapy (85% versus 73%) as compared to patients with HR-positive IDC. Patients starting with palliative chemotherapy during the first six months had a significantly shorter median residual survival thereafter as compared to those who did not (15 months versus 44 months for ILC, and 17 and 42 months for IDC, respectively). Reversely, patients treated with endocrine therapy in the first six months had a longer median residual survival compared to those who did not (37 months versus 11 months for ILC and 33 and 19 months for IDC, respectively). Our results confirm that, in addition to HR-status, histology correlates with presentation of metastatic disease by number and type of distant metastatic sites, and thereby affects treatment-decision making in real-life.

The intriguing scientific question is to determine how histological subtype influences the metastatic spread in HR-positive breast cancer. It is hypothesized that the loss of E-cadherin, a cell-cell adhesion molecule, in ILC may result in less adhesiveness of the tumor cells and therefore disseminate and infiltrate certain distant locations more easily [1].

In this HR-positive metastatic breast cancer cohort we also looked at the association of palliative systemic treatment with survival of patients with metastatic breast cancer for both histological subtypes. With the favorable presentation of distant metastasis by number and location of patients with HR-positive ILC as compared to HR-positive IDC, one would expect a more indolent disease course and a better outcome. However, we showed that for patients with HR-positive breast cancer, overall disease outcome was comparable irrespective of histology and irrespective of different treatment choices made in real-life, as discussed above. And interestingly, the efficacy of chemotherapy - once the treatment choice was made - was comparable irrespective of histology. However, treatment with palliative endocrine therapy was only associated with a favorable prognosis of patients with HR-positive ILC, whereas this was not seen in patients with HR-positive IDC, also suggesting the impact of histology over HR-status. This is in concordance with the early breast cancer setting, in which there is evidence suggesting differences in efficacy of systemic therapy between the two histological subtypes [3–5, 12].

Although there is some proof that histology could be helpful in treatment decision-making [13–15], the current guidelines do not include histological subtype as an indicator for the use of systemic treatment in general, or for a specific regimen [16, 17]. Interestingly, in the adjuvant setting, histology has been shown to be of importance when deciding between the use of breast-conserving surgery or mastectomy after neo-adjuvant chemotherapy [18]. The current study shows that histological subtype can be of predictive value for HR-positive metastatic breast cancer with regards to the effectiveness of early initiation of palliative endocrine therapy. It may be possible that in patients with HR-positive lobular breast cancer, the higher incidence of bone metastases as initial metastatic site partly accounts for the initial choice of endocrine therapy. It underlines the relevance of acknowledging a different metastatic pattern, and thereby different initial treatment choices, between breast cancer patients with ductal versus lobular histology.

Much more than in the adjuvant setting, treatment decisions in the metastatic setting are based on the observed and anticipated clinical course of the disease, which is not only determined by the tumor characteristics as described earlier. Also age, performance status, previous therapies and toxicities, comorbidity and patient and doctor preferences play a role. The complexity of this process, together with the retrospective design of our study make it impossible to identify and rule out confounding by indication. Furthermore, localization of the metastases (visceral versus bone metastasis for instance) could be related to symptomatology and thereby timing of detection, and could therefore have introduced lead time bias.

In this study patients with pure lobular carcinoma and mixed lobular carcinomas were combined for the analyses. In other studies on histological subtypes of breast cancer mixed and lobular carcinoma had similar outcome [14, 19]. Even with combining these subgroups, the proportion of HER2-positive tumors was too low to further analyze anti-HER2 therapy. In the adjuvant treatment setting the magnitude of benefit from adjuvant trastuzumab between patients with ILC and IDC was shown not to be different [20]. Another limitation of our study is that patient numbers were too small to perform analysis on specific chemotherapy regimens. Microarray analysis have demonstrated that ILC and IDC can be distinguished on the basis of genomic and expression profiles [21]. The increasing knowledge on genomic differences between ILC and IDC can help to answer questions on *in vivo* chemosensitivity. For example, topoisomerase-IIα gene amplification is a predictive biomarker for response to anthracyclines and in ILC this gene amplification is lacking, which could help understand the poor responsiveness of ILC to neo-adjuvant chemotherapy, including anthracyclines [22]. This genetic information can help guide further research and may eventually be useful in making treatment decisions for histological breast cancer subtypes.

In conclusion, to our knowledge, this is the first study that investigates the role of histological subtype in HR-positive metastatic breast cancer. Although survival was comparable for the two histological subtypes, this was achieved with different treatment strategies. As patients with HR-positive ILC were less likely to receive chemotherapy than those with HR-positive IDC, histology may be a relevant factor in treatment-decision making. For a more definite conclusion on the role of histology, we recommend to incorporate histological subtype as a stratification factor in future clinical trials.

## MATERIALS AND METHODS

### Patient selection

We identified all patients diagnosed with metastatic breast cancer between 2007–2009 in eight hospitals in the South-East of the Netherlands. All patients with metastatic breast cancer were selected irrespective of the date of primary breast cancer diagnosis (also including patients with *de novo* metastatic breast cancer; 18.5% of patients with IDC and 19.9% of patients with ILC), with the exception of patients with a diagnosis of primary breast cancer before 1990, due to limitations in the availability of data on the primary tumor and initial treatment.

Histology was classified according to the International Classification of Diseases for Oncology (ICD-O) [23]. ILC and mixed histology were defined as code 8520 and 8522. IDC was defined as code 8500. From the total of 815 metastatic breast cancer patients, we excluded 76 patients with either unknown histology or histological subtypes other than IDC or ILC. Of the remaining 739 metastatic breast cancer patients, we excluded 171 patients with HR-negative tumors (27% of patients with IDC and 8% of patients with ILC) in order to rule out the impact of HR status. In total, our study population consisted of 568 patients divided in two groups based on histology; one group of 437 patients with IDC and the other group of 131 patients with ILC.

### Data collection

Information was collected on patient and tumor characteristics, treatment and outcome. Tumors were characterized by the sixth edition of the TNM classification of malignant tumors [24] and Scarff Bloom Richardson (SBR) histological grading [25]. Estrogen receptor (ER) and progesterone receptor (PR) positivity was defined as positive nuclear staining of ≥ 10%. HER2 positivity was defined as immunohistochemistry score of 3+ or 2+ with positive FISH. In case of missing HER2 status a dedicated pathologist centrally reviewed missing data when material was available. Initial sites of metastasis were categorized as: bone, visceral (including lung, liver, pleural, peritoneal, pericardial and lymphangitic carcinomatosis), brain (including leptomeningeal and CNS), skin and lymph nodes, and multiple metastases (more than one of the metastatic sites).

### Statistical analysis

Baseline characteristics between the two histological groups were compared using chi-square tests for categorical variables and Wilcoxon rank sum tests for continuous variables.

Metastatic-free interval was defined as time between date of primary diagnosis and date of first distant metastasis. Overall survival after diagnosis of metastatic breast cancer was defined as time between date of first distant metastasis and date of death. Survival curves and time to first palliative systemic therapy (either chemotherapy or endocrine therapy) were estimated using the Kaplan-Meier method and compared using log-rank tests. All patients still alive were censored at the date of last follow-up of each individual patient. Patients who died without palliative therapy were censored at the date of death in the analysis of time to first palliative therapy.

To explore the association of palliative systemic therapy with the survival of patients with metastatic breast cancer for both histological subtypes a Cox proportional hazards model was performed with palliative chemotherapy and endocrine therapy as a time-dependent covariate, since the administration of treatment can change over time and is dependent on the time available for each patient to receive the treatment. We did not explore the association between palliative targeted therapy, such as trastuzumab and bevacizumab, and survival since the number of patients with IDC and ILC receiving targeted therapy was very low. Since all these patients received targeted therapy with chemotherapy, these patients were included in the analysis on initial chemotherapy. In addition, the landmark method was used to estimate survival after a specific time-point, the so-called residual survival [26]. As we were interested to learn about the obtained survival in relation to the initial palliative treatment choices, we chose six months after diagnosis of metastatic breast cancer as landmark. Consequently, patients who already died within 6 months were excluded for the residual survival curves.

All analyses were performed using SAS version 9.2. All reported *P*-values are two-sided and *P*-value ≤ 0.05 was considered statistically significant.
